# A novel similarity measurement for triangular cloud models based on dual consideration of shape and distance

**DOI:** 10.7717/peerj-cs.1506

**Published:** 2023-08-09

**Authors:** Jianjun Yang, Jiahao Han, Qilin Wan, Shanshan Xing, Fei Chen

**Affiliations:** 1 School of Automobile and Transportation, Xihua University, Chengdu, Sichuan Province, China; 2 Provincial Engineering Research Center for New Energy Vehicle Intelligent Control and Simulation Test Technology of Sichuan, Xihua University, Chendu, Sichuan Province, China; 3 Vehicle Measurement, Control and Safety Key Laboratory of Sichuan Province, Xihua University, Chendu, Sichuan Province, China

**Keywords:** Cloud model, Expectation curve, Similarity measurement, Triangular fuzzy number, EW-type closeness, Cloud model variance

## Abstract

It is important to be able to measure the similarity between two uncertain concepts for many real-life AI applications, such as image retrieval, collaborative filtering, risk assessment, and data clustering. Cloud models are important cognitive computing models that show promise in measuring the similarity of uncertain concepts. Here, we aim to address the shortcomings of existing cloud model similarity measurement algorithms, such as poor discrimination ability and unstable measurement results. We propose an EPTCM algorithm based on the triangular fuzzy number *EW*-type closeness and cloud drop variance, considering the shape and distance similarities of existing cloud models. The experimental results show that the EPTCM algorithm has good recognition and classification accuracy and is more accurate than the existing Likeness comparing method (LICM), overlap-based expectation curve (OECM), fuzzy distance-based similarity (FDCM) and multidimensional similarity cloud model (MSCM) methods. The experimental results also demonstrate that the EPTCM algorithm has successfully overcome the shortcomings of existing algorithms. In summary, the EPTCM method proposed here is effective and feasible to implement.

## Introduction

Natural language is a valuable tool for human communication and thinking. However, there can be great uncertainty in the use of language, which can be summarized by the concepts of randomness and fuzziness (Li & Du, 2017). The research on natural language processing is both challenging and poetic. Artificial intelligence is also marked by ambiguity (Müller et al., 2022), especially in the era of big data. Although the development of information transmission and storage technology has improved big data processing, it is still impossible to obtain a complete, real-time picture of all the data. Li, Di & Li (2000) put forward the cloud model theory in the early 1990s, which integrates fuzziness and randomness, realizes the mutual conversion between qualitative concepts and quantitative representations, and is intuitive and universal. After several years of exploration and development, the cloud model has become completer and more universal (Wang, Li & Yang, 2019). The cloud model has been successfully applied and developed in many fields, such as in the statistical representation of engineering parameters (Chen et al., 2020; Luo et al., 2022), system evaluation and decision-making (Tong & Srivastava, 2022; Su & Yu, 2020; Wu et al., 2020), data mining (Shehab, Badawy & Ali, 2022; Zarinbal, Zarandi & Turksen, 2014), image processing (Li, Li & Du, 2018; Tversky & Kahneman, 1992), and decision making problems (Yu et al., 2021; Wang, Huang & Cai, 2020; Zhou, Chen & Ming, 2022). It should be noted that the practical applications of cloud model theory (such as data mining and decision analysis) all involve the similarity measurement (Zhang, Zhao & Li, 2004). Therefore, the similarity measurement will directly influence the actual application of the cloud model theory.

The comparison between the similarities of cloud model applications is of great interest to researchers (Li, Wang & Yang, 2019). Cloud models express the uncertainty of data intuitively and provide a method with which to analyze qualitative concepts similarly to that of human cognition. The randomness and fuzziness of the cloud model make it more advantageous in dealing with uncertain problems such as data clustering (Sheng et al., 2019), data classification (Wang et al., 2021), and similarity searches (Luo et al., 2022). Cloud models have been developed and improved over time, resulting in the development of various similarity measurement methods. Zhang et al. (2007) viewed the digital features of two cloud models as elements of two vectors and characterized the similarity of the cloud models by the cosine angle of the two vectors (LICM). Li, Guo & Qiu (2011) proposed the area proportional method (expectation-based cloud mode, ECM) based on the expectation curve. This method uses the intersection area surrounded by the expectation curve and the horizontal axis of two cloud models to represent similar components, resulting in the similarity of the cloud models. Inspired by the relationship between the Gaussian distribution and GCM, researchers have utilized the distance of probability distributions to determine the Kullback–Leibler divergence (KLD) (Xu & Wang, 2017), earth-movers’ distance (EMD) (Yang, Wang & Li, 2016), and the square root of the Jensen–Shannon divergence (Yang et al., 2018), to describe the concept of drift (EMDCM) which is reflected by the distance between two cloud models. Wang et al. (2018) defined a new measure of fuzzy distance for model clouds based on the *α*-cuts and they proposed a new cloud model similarity measurement method using the fuzzy distance measurements (fuzzy distance-based similarity, FDCM). Yan et al. (2019) used the overlap-based expectation curve of cloud model (OECM) algorithm as a measurement method to measure the similarity of cloud models. In this algorithm, the overlapping degree is used to describe the overlapping part of two clouds, and the overlapping part is transformed into the similarity of cloud models by using the membership degree of “3*En*” boundary and the intersection of two clouds. Li et al. (2020) proposed a cloud model similarity measurement method (UDCM) based on uncertain distribution. Zhang et al. (2021) put forward a new similarity measurement method (MSCM) for multi-dimensional cloud models based on fuzzy similarity principle. Luo et al. (2022) proposed a new structural damage identification method (MCM) based on a cloud model similarity measurement of response surface model updating. A brief summary is given in Table 1 to illustrate the shortcomings of the existing methods.

**Table 1:** A summary of previous cloud model similarity metrics.

**Literature**	**Publication year**	**Main contents**	**Limitations**
Zhang et al.	2007	LICM method	• The discrimination of the measurement results was low;
			• When there is a large difference between the numerical features of the cloud model, the calculated similarity error is larger.
Li, Guo & Qiu	2011	ECM method	• The method ignores the role of hyper-entropy (*He*) for cloud models, and the metric results are generally different from human cognition;
			• The calculation steps are tedious and the arithmetic is complicated.
Yang et al.	2018	EMDCM method	• The discrimination of the measurement results was low;
			• The method do not find the difference between two different concepts in some special situations due to neglecting the variation of hyper-entropy (*He*);
			• Moreover, it only have partial interpretability due to the absence of relationship between entropy and hyper-entropy (Li, Wang & Yang, 2019).
Wang et al.	2018	FDCM method	• The method still has a complex arithmetic process and is costly to run on the CPU;
			• Measurement results remain unstable;
			• The threshold (*δ*) of cloud droplets is difficult to determine.
Yan et al.	2019	OECM method	• The discrimination of the measurement results is not good;
			• The algorithm only considers the overlap between cloud models, and does not consider the shape similarity of cloud models, which can only be partially explained.
Li et al.	2020	UDCM method	• This method still has integral operations and consumes a large amount of CPU runtime;
			• The calculation results are still influenced by the number of cloud droplets and the number of experiments.
Zhang et al.	2021	MSCM method	• The algorithm can fail in some special cases;
			• For example, when the two cloud expectation (*Ex*) are equal, the metric result will be constant at 1. It ignores the role of entropy (*En*) and hyper-entropy (*He*), which is inconsistent with human subjective cognition and has loopholes.
Luo et al.	2022	MCM method	• Although hyper-entropy is considered in MCM, it will fail when hyper-entropy is very large;
			• The calculation steps are tedious and the arithmetic is complicated.

Currently, there is no consensus on how to evaluate the similarity measurement method of cloud models. However, a good cloud concept similarity metric algorithm needs to be stable and efficient, and able to highlight the differences between the different types of clouds. It should ensure greater differentiation and guarantee correct similarity conclusions. In addition, a similarity metric for cloud models with good performance should be generic.

In order to solve the problems of existing cloud model similarity metrics, this study aims to propose a new cloud model similarity measurement method using the triangle cloud model (Gong, Dai & Hu, 2016), an extended model of the normal cloud model, as the research object. The triangular cloud model similarity (PCM), based on cloud drop variance, is proposed as the shape similarity of two groups of cloud models. It is combined with triangular cloud model distance similarity (ETCM) based on *EW*-type closeness (Bao & Bai, 2018). Extra consideration is given to the distance and shape similarity of the cloud model and this method was shown to return better discrimination results. The experimental results show that the discrimination is higher. The simulation results show that the measurement results obtained by EPTCM method are consistent with people’s intuitive impression. This can prove that this method is reasonable. It can be analyzed that the process in this paper can better distinguish different types of cloud models.

### Definitions and notions

Here, we provide definitions, relationships, and necessary lemmas for the normal cloud, triangular cloud model, and triangular fuzzy numbers. We then describe the variance of triangular cloud model and *EW*-type closeness.

**Definition 1.** Let *U* be a non-empty infinite set expressed by an accurate numerical value, and *C* is a qualitative concept on *U*. If there is an accurate numerical value *x* ∈*U*, and the mapping $y={\mu }_{C} \left( x \right) \in \left[ 0,1 \right] $ of *x* to *C* is a random number with a stable law, then the distribution of (*x*, *y*) on the universe *U* is called a cloud, and each (*x*, *y*) is called a cloud drop (Li, Han & Shi, 1998).

**Definition 2.** The three characteristic parameters (*Ex*, *En*, *He*) of the cloud model are the quantitative embodiment of its qualitative concept. The expectation (*Ex*) of the cloud is the representation of the expected value of the cloud in the non-empty infinite set expressed and it is also the center of gravity corresponding to the maximum value of the membership degree *Y*; entropy (*En*) is a measure of the uncertainty of cloud model, which reflects the expected dispersion of cloud droplets and the fuzziness of cloud model data. Hyper-entropy (*He*) is the entropy of *En*, which is a measure of the uncertainty of cloud model entropy. Its value can represent the thickness of cloud, reflecting the randomness of cloud model data.

**Definition 3.** If the random variable *x* satisfies *x* ∼ *N*(*Ex*, *En*^‘^^2^), where *En*^‘^ ∼ *N*(*En*, *He*^2^), and the certainty of *x* to the qualitative concept satisfies:

(1) \begin{eqnarray*}{\mu }_{C}(x)=\exp \nolimits (- \frac{(x-Ex)^{2}}{2(E{n}^{{^{\prime}}})^{2}} )\end{eqnarray*}

then that distribution of *x* on the non-empty infinite set *U* is normal cloud (Li, Han & Shi, 1998).

**Definition 4.** If the random variable *x* satisfies *x* ∼ *N*(*Ex*, *En*^‘^^2^), where *En*^‘^ ∼ *N*(*En*, *He*^2^), and the certainty of *x* to the qualitative concept satisfies:

(2) \begin{eqnarray*}{\mu }_{C}(x)= \left\{ \begin{array}{@{}l@{}} \displaystyle \frac{x- \left( Ex-3E{n}^{{^{\prime}}} \right) }{3E{n}^{{^{\prime}}}} ,\quad x\lt Ex\\ \displaystyle 1- \frac{x-Ex}{3E{n}^{{^{\prime}}}} ,\quad x\geq Ex \end{array} \right. \end{eqnarray*}

then that distribution of *x* on the non-empty infinite set *U* is triangle cloud.

**Definition 5.** If the random variable *x* satisfies *x* ∼ *N*(*Ex*, *En*^‘^^2^), where *En*^‘^ ∼ *N*(*En*, *He*^2^), and *En* ≠0. At this time, the Eq. (3) exists:

(3) \begin{eqnarray*}y= \left\{ \begin{array}{@{}l@{}} \displaystyle \frac{x- \left( Ex-3En \right) }{3En} ,\quad x\lt Ex\\ \displaystyle 1- \frac{x-Ex}{3En} ,\quad x\geq Ex \end{array} \right. \end{eqnarray*}

then *y* is called the expected curve of triangular cloud. The expected curve is obtained from the distribution law of cloud droplets in the horizontal direction. The expected curve can intuitively describe the shape characteristics of triangular cloud and all cloud droplets fluctuate randomly around the expected curve.

**Definition 6.** Fuzzy numbers are convex fuzzy sets defined on real numbers *R* (Wu & Zhao, 2008). For a certain fuzzy number, its membership degree satisfies Eq. (4):

(4) \begin{eqnarray*}F \left( x \right) = \left\{ \begin{array}{@{}l@{}} \displaystyle \frac{x-{r}^{l}}{{r}^{m}-{r}^{l}} ,\quad {r}^{l}\leq x\leq {r}^{m}\\ \displaystyle \frac{x-{r}^{u}}{{r}^{m}-{r}^{u}} ,\quad {r}^{m}\leq x\leq {r}^{u}\\ \displaystyle \begin{array}{@{}l@{}} \displaystyle 0,\quad \text{other} \end{array} \end{array} \right. \end{eqnarray*}

then $\tilde {r}=({r}^{l},{r}^{m},{r}^{n})$ is called triangular fuzzy number. The membership function of $\tilde {r}$ is $F \left( x \right) :R\rightarrow \left[ 0,1 \right] $, where *x* ∈ *R* and *R* is a real number field. *r*^*l*^, *r*^*m*^, *r*^*u*^ are the lower bound, median, and upper bound of triangular fuzzy numbers, respectively, and *r*^*l*^ ≤ *r*^*m*^ ≤ *r*^*u*^. When they are equal, $\tilde {r}$ degenerate into real values.

**Figure 1:**
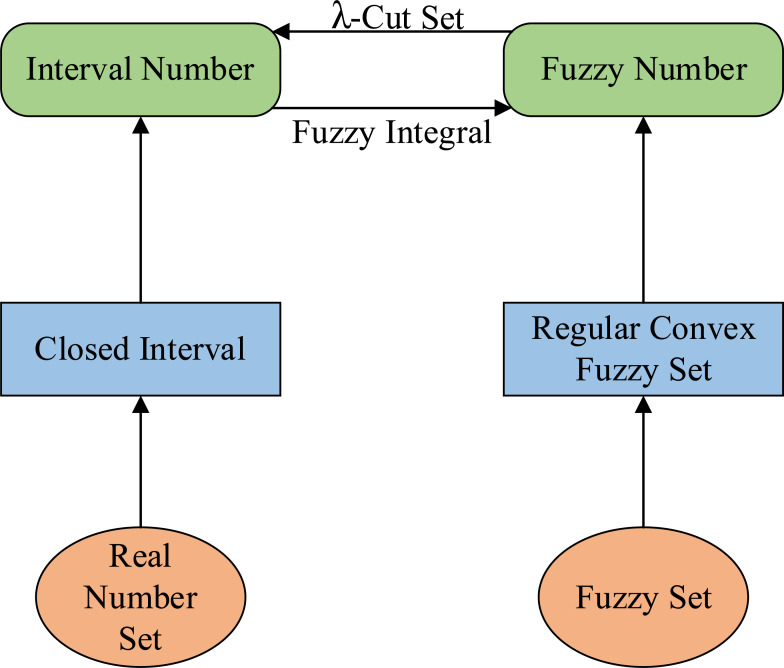
The relationship between fuzzy number and interval number.

**Definition 7.** For $a_{\text{_}},\overline{a}\subseteq R$, and $a_{\text{_}}\leq \overline{a}$, then $a= \left[ a_{\text{_}},\overline{a} \right] $ is called interval number. The relationship between fuzzy numbers and interval numbers is shown in Fig. 1. The whole number of intervals is recorded as [*R* ]. for *a* ∈ [*R*], the Eq. (5) exists:

(5) \begin{eqnarray*}E \left( a \right) = \frac{\overline{a}+a_{\text{_}}}{2} ,\,\,\,\,\,\,W \left( a \right) = \frac{\overline{a}-a_{\text{_}}}{2} \end{eqnarray*}

*E*(*a*) and *W*(*a*) are respectively the expected value and width of interval number *a* (Bao, Peng & Zhao, 2013).

**Definition 8.** For *u* ∈ *F*_0_, *F*_0_ is a fuzzy number space (Bao & Bai, 2018). The *r*-cut set with fuzzy number *u* is a closed interval, as shown in Eq. (6):

(6) \begin{eqnarray*}{ \left[ u \right] }^{r}= \left[ u_{\text{_}} \left( r \right) ,\overline{u} \left( r \right) \right] ,\,\,\,r\in \left[ 0,1 \right] .\end{eqnarray*}

**Figure 2:**
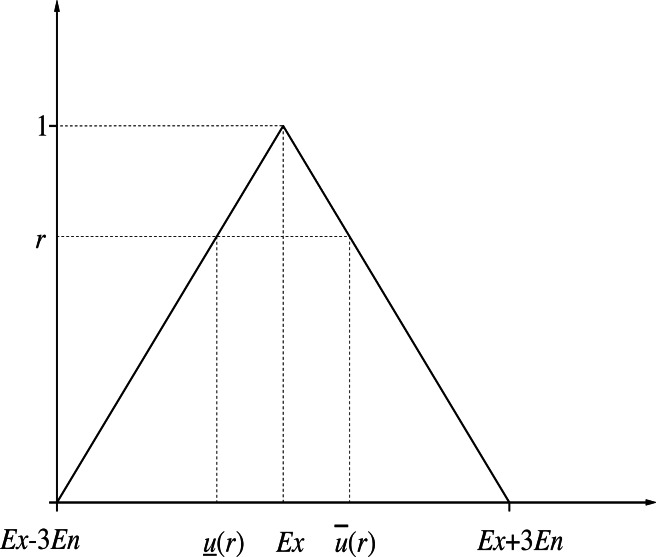
The r-cut interval number of triangular numbers.

The *r*-cut interval number of triangular numbers is shown in Fig. 2.

The order relation on *F*_0_ is defined as:

*u* ≤ *v*, and if and only if for any $r\in \left[ 0,1 \right] ,\overline{u} \left( r \right) \leq \overline{v} \left( r \right) $ and . $u_{\text{_}} \left( r \right) \leq v_{\text{_}} \left( r \right) $.

*u* ≥ *v*, and if and only if for any $r\in \left[ 0,1 \right] ,\overline{u} \left( r \right) \geq \overline{v} \left( r \right) $ and $u_{\text{_}} \left( r \right) \geq v_{\text{_}} \left( r \right) $.

Definition 8 can be regarded as a bridge between fuzzy numbers and interval numbers, and it is also the theoretical basis for the transformation of interval number closeness to fuzzy number closeness in *EW*-type closeness.

**Lemma 1.** If the random variable *x* satisfies *x* ∼ *N*(*Ex*, *En*′^2^), where *En*′ ∼ *N*(*En*, *He*^2^), and *En* ≠ 0.

(7) \begin{eqnarray*}D(x)=E{n}^{2}+H{e}^{2}\end{eqnarray*}

The *D* (*x*) is called the variance of cloud droplets in the cloud model. *He* determines the thickness of clouds, and *En* determines the dispersion degree of cloud droplets. The larger the difference between *He* and *En*, the smaller the shape similarity between two clouds model.

**Lemma 2.** Set function: (Bao & Bai, 2018)

(8) \begin{eqnarray*}g(x)= \frac{1}{1+{x}^{n}} ,n\gt 0,x\in [0,+\infty )\end{eqnarray*}

The mapping *f*: $ \left[ R \right] \times \left[ R \right] \rightarrow [0,+\infty )$ is defined as:

(9) \begin{eqnarray*}f \left( u,v \right) ={ \left( {|}E \left( u \right) -E \left( v \right) {{|}}^{p}+ \frac{1}{3} {|}W \left( u \right) -W \left( v \right) {{|}}^{p} \right) }^{ \frac{1}{p} },\,p\geq 1,\forall u,\,v\in \left[ R \right] \end{eqnarray*}

then $g \left( f \left( u,v \right) \right) ={n}_{EW} \left( u,v \right) $ is the closeness of the interval number *u* and *v*.

**Lemma 3.** Let the mapping ${N}_{EW}^{p}:{F}_{0}\times {F}_{0}\rightarrow \left[ 0,1 \right] $ be defined as:

(10) \begin{eqnarray*}{N}_{EW}^{p} \left( u,v \right) ={ \left( \int \nolimits \nolimits _{0}^{1}({n}_{EW}([u]^{r},[v]^{r}))^{p}dr \right) }^{ \frac{1}{p} },p\geq 1,\forall u,v\in {F}_{0}\end{eqnarray*}

then ${N}_{EW}^{p} \left( u,v \right) $ is called *EW*-type closeness of triangular fuzzy numbers *u* and *v* (Bao & Bai, 2018).

## Materials & Methods

### A novel similarity measure for triangular cloud model

Many studies consider only one aspect to measure the similarity of cloud models, therefore, these methods have some shortcomings. In fact, the similarity of cloud models can be observed from two aspects: shape and distance. By combining these two perspectives with scientific methods, a more complete similarity measurement method for cloud models can be obtained. The EPTCM method proposed here is formed from this perspective. The EPTCM method consists of a combination of two methods (the ETCM and PCM methods). Since the ETCM method introduces only *Ex* and *En*, it is considered as the distance similarity between the cloud models. However, the PCM method is exactly related to the shape of the two cloud models (only *En* and *He* are considered). After establishing these two methods, a scientific empowerment method is designed in this article to combine these two methods scientifically. The ETCM method and PCM method are combined by empowerment to obtain the final EPTCM method.

### Similarity measure of expected curve of cloud model based on *EW*-type closeness

Fuzzy closeness is an important concept for triangular fuzzy numbers (Jiang et al., 2019). Compared with the traditional Hausdorff-distance (Hossein-Abad, Shabanian & Kazerouni, 2020) and P-distance (You & Yan, 2017) formulas, the *EW*-type distance formula $({d}_{EW}^{P})$ (Bao, Peng & Zhao, 2013) used in *EW*-type closeness (Bao & Bai, 2018) considers the expected difference of two interval numbers and takes into account the difference of their widths. The simulation results from Bao, Peng & Zhao (2013) show that this method describes the distance of interval numbers more comprehensively and meticulously, and the utilization rate of information is greatly improved. Compared with the traditional exponential closeness (Eq. (11)), *EW*-type closeness introduces the degree of the interval number closeness to participate in the calculation. Bao & Bai (2018) show that *EW*-type closeness has better discrimination and practicability.

(11) \begin{eqnarray*}d(A,B)={e}^{-\sqrt[p]{{|}{a}^{l}-{b}^{l}{{|}}^{p}+{|}{a}^{m}-{b}^{m}{{|}}^{p}+{|}{a}^{r}-{b}^{r}{{|}}^{p}/q}}\end{eqnarray*}

**Figure 3:**
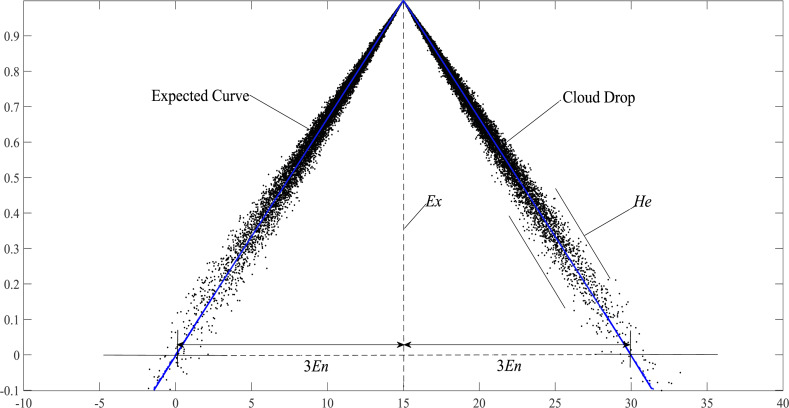
Schematic diagram of the expected curve structure parameters of the cloud model.

According to the “3*En*” rule of the triangle cloud model (Li, Han & Shi, 1998), more than 90% of cloud droplets fall in the range of [*Ex* − 3*En*, *Ex* + 3*En*]. Therefore, when calculating the similarity of the triangular cloud model, we only need to consider the cloud droplets in this range and the expected curve. As shown in Fig. 3, the forward triangle cloud “3*En*” rule is introduced to the expected curve in Definition 5, and the transformed curve formula:

(12) \begin{eqnarray*}{y}^{{^{\prime}}}(x)= \left\{ \begin{array}{@{}cc@{}} \displaystyle \frac{x-(Ex-3En)}{Ex-(Ex-3En)} ,&\displaystyle (Ex-3En)\leq x\leq Ex\\ \displaystyle 1,&\displaystyle x=Ex\\ \displaystyle \frac{(Ex+3En)-x}{Ex+3En-Ex} ,&\displaystyle Ex\,\leq x\leq (Ex+3En)\\ \displaystyle 0,&\displaystyle \text{other} \end{array} \right. \end{eqnarray*}

Equation (12) conforms to the definition of the triangular fuzzy number in Definition 6 and is denoted as *y*′ =  < *Ex* − 3*En*, *Ex*, *Ex* + 3*En* >. Therefore, *EW*-type closeness can be applied to *y*′. If *Ex* − (*Ex* − 3*En*) = (*Ex* + 3*En*) − *Ex* = 3*En*, *y*′ is called the symmetric triangular fuzzy number based on the expected curve (Hwang & Yang, 2007), it is noted as ${\tilde {y}}^{{}^{{^{\prime}}}}=(Ex,3En)_{T}$, where *Ex* and 3*En* are the expectation and ambiguity (also called width) of the triangular fuzzy number based on the expected curve, respectively.

According to Definitions 7 and 8, the upper bound and lower bound of the r-truncated closed interval ${ \left[ u \right] }^{r}$ of the expected curve can be obtained from triangular fuzzy number *y*′ =  < *Ex* − 3*En*, *Ex*, *Ex* + 3*En* >:

(13) \begin{eqnarray*} \left\{ \begin{array}{@{}c@{}} \displaystyle Upper\,bound:\overline{u} \left( r \right) =Ex+3En \left( 1-r \right) \\ \displaystyle Lower\,bound:u_{\text{_}} \left( r \right) =Ex-3En \left( 1-r \right) \end{array} \right. \end{eqnarray*}

The expectation and width of interval *u* are:

(14) \begin{eqnarray*} \left\{ \begin{array}{@{}c@{}} \displaystyle E \left( u \right) = \frac{\overline{u}+u_{\text{_}}}{2} =Ex\\ \displaystyle W \left( u \right) = \frac{\overline{u}-u_{\text{_}}}{2} =3En \left( 1-r \right) \end{array} \right. \end{eqnarray*}

Therefore, the triangular fuzzy number *v* = 〈*Ex*′ − 3*En*′, *Ex*′, *Ex*′ + 3*En*′〉 of another group of cloud expectation curves is set. The similarity of the expectation curves of the two groups of cloud models based on *EW*-type closeness can be obtained from Lemma 3.

(15) \begin{eqnarray*}Sim \left( ETCM \right) ={N}_{EW}^{p} \left( u,v \right) ={ \left( \int \nolimits \nolimits _{0}^{1}{ \left( {n}_{EW} \left( { \left[ u \right] }^{r},{ \left[ v \right] }^{r} \right) \right) }^{p}dr \right) }^{ \frac{1}{p} },\quad p\geq 1.\end{eqnarray*}

The ETCM algorithm constructs two sets of cloud model expectation curves under the restriction of “3*En*” principle into two triangular fuzzy numbers. The *EW*-type closeness can be used to calculate the similarity of the two triangular fuzzy numbers under *r* ∈ [0, 1]. Because the ETCM method does not introduce *He* into the calculation, it was considered to be the distance similarity of the triangular cloud model. The larger the *Sim* (ETCM) value, the higher the distance similarity between the two triangular cloud models, and vice versa.

### Shape similarity measurement of triangular cloud model based on cloud drop variance

All clouds can be translated to the position *x* = 0, therefore, the shape of the cloud has nothing to do with the cloud’s expectation (*Ex*). As mentioned earlier, the cloud’s *En* and *He* reflect the shape of the cloud and describe the conceptual expansion of the variables. It is clear that the basic skeleton of the cloud model shape is determined by *En* with the “3*En*” rule. In addition, the *He* controls the dispersion of the thickness or conceptual extension of the cloud. Based on the above theory, we sought to determine the relationship mathematically.

According to Lemma 1, the variance *D*(*x*) = *En*^2^ + *He*^2^ of the triangular cloud model consists of *En* and *He*. Although the variance does not consider the *Ex* (the location relationship of the cloud models) it fully reflects the shape similarity between cloud models. The greater the *En* difference between two clouds, the smaller the shape similarity between two clouds, so consider introducing the mean square error of cloud model to measure the shape similarity of cloud model. If there are two groups of triangular cloud models *C*_*i*_(*Ex*_*i*_, *En*_*i*_, *He*_*i*_) and *C*_*j*_(*Ex*_*j*_, *En*_*j*_, *He*_*j*_), their shape similarity is expressed as:

(16) \begin{eqnarray*}Sim \left( PCM \right) = \frac{\min \nolimits \left( \sqrt{D{ \left( x \right) }_{i}},\sqrt{D{ \left( x \right) }_{j}} \right) }{\max \nolimits \left( \sqrt{D{ \left( x \right) }_{i}},\sqrt{D{ \left( x \right) }_{j}} \right) } \end{eqnarray*}

**Figure 4:**
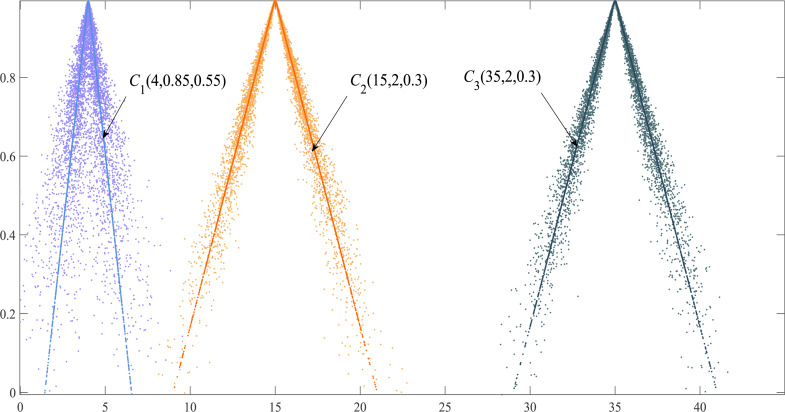
Cloud droplet distribution and the expected curve of three cloud models.

Regardless of the difference between the *Ex* of the two cloud models, if the *En* and *He* of the two clouds are equal, their shape similarity *Sim* (PCM) = 1. Although the *Ex* of triangular cloud models *C*_2_ and *C*_3_ are different, the shapes of the two clouds are consistent (Fig. 4). The PCM method has better authenticity and timeliness compared with the similarity method based on the maximum boundary curve of the cloud model in Li, Guo & Qiu (2011) whose model exaggerates the proportion of *He* in calculating shape similarity.

### The integrated similarity measurement of the triangular cloud model

As previously mentioned, the ETCM method does not consider the influence of *He*, while the PCM method does not consider the influence of cloud model *Ex*. We defined a weighted calculation method, which combines the two methods to enrich the completeness and authenticity of cloud model similarity in order to create an integrated approach that incorporated the three main characteristic parameters of the cloud model (*Ex*, *En*, *He*). Referring to the analytic hierarchy process (AHP) and entropy weight method proposed in the reference (Ruan et al., 2017), we defined a method to determine the similarity weight of the integrated cloud model in Eq. (17).

(17) \begin{eqnarray*} \left\{ \begin{array}{@{}l@{}} \displaystyle d{ \left( \alpha ,\beta \right) }^{2}=\sqrt{{ \left( Sim \left( ETCM \right) -Sim \left( PCM \right) \right) }^{2}}\\ \displaystyle \,d{ \left( \alpha ,\beta \right) }^{2}={ \left( \alpha -\beta \right) }^{2}\\ \displaystyle \alpha +\beta =1\\ \displaystyle \alpha \geq \beta . \end{array} \right. \end{eqnarray*}

The calculated weight coefficients (*α* and *β*) were used to weight the triangular cloud ETCM method and PCM method, respectively. Thus, a similarity measurement algorithm for triangular cloud models based on the dual consideration of shape and distance is defined. As shown in Eq. (18):

(18) \begin{eqnarray*}Sim \left( \text{EPTCM} \right) =\alpha Sim \left( \text{ETCM} \right) +\beta Sim \left( \text{PCM} \right) \end{eqnarray*}

Equation (18) is the final expression of the proposed EPTCM method. The complete computational procedure of the EPTCM algorithm is shown in Algorithm 1. In order to verify that the EPTCM method was feasible and effective, scientific simulation experiments were conducted.

**Table:** 

**Algorithm 1.** A cloud model similarity metric algorithm based on *EW*-type closeness and cloud droplet variance. (**MATLAB**)
**Input**: Two sets of cloud models: *C*_*i*_(*Ex*_*i*_, *En*_*i*_, *He*_*i*_) and *C*_*j*_(*Ex*_*j*_, *En*_*j*_, *He*_*j*_).
**Output**: The $Sim \left( \text{EPTCM} \right) $ of the two sets of cloud models.
**1** function [EPTCM] = EPTCM(PO,PN)
**2** z = 1;
**3** t = 1;
**4** Ex1 =PO(1);
**5** En1 =PO(2);
**6** He1 =PO(3);
**7** Ex2 =PN(1);
**8** En2 =PN(2);
**9** He2 =PN(3);
%The two sets of cloud model expectation curves are expressed in the form of triangular fuzzy numbers.
**10** symsr;
**11** ux1 = Ex1 − 3∗En1∗(1 − r);
**12** us1 = Ex1 + 3∗En1∗(1 − r);
**13** ux2 = Ex2 − 3∗En2∗(1 − r);
**14** us2 = Ex2 + 3∗En2∗(1 − r);
% The expectation values *E* (*u*) and *E* (*v*) and the widths *W* (*u*) and *W* (*v*) of the two triangular blurred numbers are calculated, respectively.
**15** Eu1 = (ux1 + us1)/2;
**16** Eu2 = (ux2 + us2)/2;
**17** Wu1 = (us1 − ux1)/2;
**18** Wu2 = (us2 − ux2)/2;
% Calculate the difference between the expected value (Δ*E*) of the two triangular cloud models, and the difference (Δ*W*) in width. Substitute into Eqs. (8) and (9) to obtain the interval number closeness (*n*_*EW*_(*u*, *v*)).
**19** $\mathrm{N1}=@(\mathrm{r})1/(1+((((\mathrm{abs}(\mathrm{Eu1}-\mathrm{Eu2}))\hat {}\mathrm{z}+((\mathrm{abs}(\mathrm{Wu1}-\mathrm{Wu2}))\hat {}\mathrm{z})/3))\hat {}(1/\mathrm{z}))\hat {}\mathrm{t})$;
% According to Eq. (15), the obtained interval number closeness (*n*_*EW*_(*u*, *v*)) is converted to *EW*-type closeness. That is *Sim* (ETCM).
**20** ETCM = int(N1, r, 0, 1);
**21** eval(ETCM);
% Calculate the mean variance of the two sets of cloud models. Calculate the distance similarity *Sim* (PCM) of two groups of cloud models by Eq. (16).
**22** $\mathrm{PCM}=(\mathrm{min}(\mathrm{sqrt}(\mathrm{En1}\hat {}2+\mathrm{He1}\hat {}2),\mathrm{sqrt}(\mathrm{En2}\hat {}2+\mathrm{He2}\hat {}2))/\mathrm{max}(\mathrm{sqrt}(\mathrm{En1}\hat {}2+\mathrm{He1}\hat {}2),\mathrm{sqrt}(\mathrm{En2}\hat {}2+\mathrm{He2}\hat {}2)))$;
% Calculate the weight coefficients of ETCM and PCM methods by Eq. (17). The final EPTCM algorithm is obtained after the weighting calculation of the two. As shown in Eq. (18).
**23** syms B;
**24** [B] =solve((1-2*B) $\hat {}$2-sqrt((ETCM-PCM) $\hat {}$2) = =0);
**25** B =vpa(B);
**26** A =B(1);
**27** if(A<B(2))
**28** C =A;
**29** A =B(2);
**30** B =C;
**31** end
**32** EPTCM =A*ETCM+B*PCM;
**33** eval(EPTCM);
**34** end

## Experiments and Results

Here, we verify the feasibility and effectiveness of the proposed EPTCM method. Simulation experiments and time series classification test experiments are conducted respectively by using MATLAB software.

### Cloud model discrimination simulation experiment

Four classical cloud models are given in Zhang et al. (2007) using the collaborative filtering algorithm as follows:

\begin{eqnarray*} \left[ \begin{array}{@{}l@{}} \displaystyle {C}_{1} \left( 1.5,0.62666,0.33900 \right) \\ \displaystyle {C}_{2} \left( 4.6,0.60159,0.30862 \right) \\ \displaystyle {C}_{3} \left( 4.4,0.75199,0.27676 \right) \\ \displaystyle {C}_{4} \left( 1.6,0.60159,0.30862 \right) \end{array} \right] \end{eqnarray*}

These four groups of classical cloud models are used to perform simulation experiments as shown in Li, Guo & Qiu (2011) and Yu et al. (2021). In order to verify the advantages of the proposed EPTCM method over the existing methods (LICM, FDCM, OECM, MSCM), the four groups of classical cloud models were also used for our simulation experiments.

**Figure 5:**
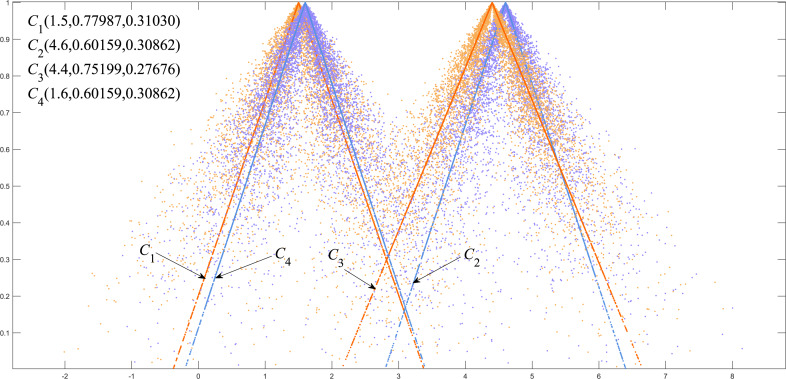
Visualization of four triangle cloud models and their expected curves.

**Figure 6:**
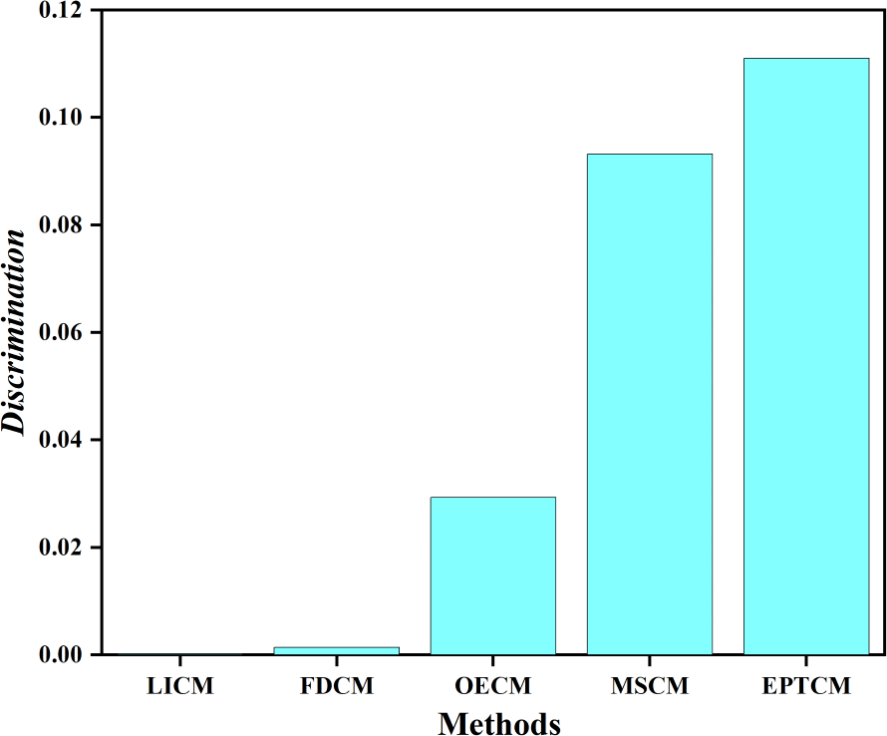
*Discrimination* results.

We classified *C*_1_, *C*_4_ as group A and *C*_2_, *C*_3_ as group B. Figure 5 shows that there is a big gap between the cloud models of group A and group B in terms of distance and shape similarity. However, within the group, the distance and shape similarity are slightly different. Therefore, the better similarity measurement method may more accurately reflect the intragroup differences between group A and group B. Equation (19) was used as the basis to measure the discrimination ability of the cloud model similarity measurement method. The following uses the above EPTCM method to measure the similarity of cloud models among these four cloud models. The EPTCM method was to compare and analyze the results with traditional methods such as LICM, OECM, MSCM and FDCM. The calculation results are shown in Table 2. The comparison chart of *Discrimination* of the five methods is presented in Fig. 6. In order to reduce the time complexity in the calculation process: *n* = 1, *p* = 1.

(19) \begin{eqnarray*}Discrimination= \left\vert Sim({C}_{1},{C}_{4})-Sim({C}_{2},{C}_{3}) \right\vert \end{eqnarray*}

**Table 2:** Measurement results of similarity of cloud models of FDCM, OECM, LICM, MSCM and EPTCM methods. The data corresponding to Formula 19 has been indicated in bold.

**Similarity**	LICM	FDCM	OECM	MSCM	EPTCM
$Sim \left( {C}_{1},{C}_{2} \right) $	0.9561	0.4850	0.0049	0	0.2996
$Sim \left( {C}_{1},{C}_{3} \right) $	0.9648	0.5068	0.0297	0	0.3167
***Sim***(***C***_**1**_**,*C***_**4**_)	**0.9990**	**0.9674**	**0.9696**	**0.8883**	**0.9183**
$Sim \left( {C}_{\mathbf{2}}\mathbf{,}{C}_{\mathbf{3}} \right) $	**0.9992**	**0.9778**	**0.9403**	**0.7951**	**0.8073**
$Sim \left( {C}_{2},{C}_{4} \right) $	0.9679	NaN	0.0057	0	0.3002
$Sim \left( {C}_{3},{C}_{4} \right) $	0.9755	0.5325	0.0335	0	0.3268

### Experiment on time series classification accuracy based on synthetic control chart dataset

#### Details of the synthetic control chart dataset

The time series represents the state of an object across different time periods and arranges them according to their time of occurrence, thus generating a data series. Time series is often used in engineering data classification, prediction, and clustering (Alcock & Manolopoulos, 1999). Here, the synthetic control chart dataset (SYNDATA) (Pham & Chan, 1998), a commonly used time series dataset in the UCI knowledge discovery database, was used to test the classification accuracy. The SYNDATA dataset contains 600 control chart samples synthesized by the processes by Alcock and Manolopoulos in 1999 (Pham & Chan, 1998). This dataset was chosen because it contains the time series’ of multiple trends, which are volatile and complex and can be a good test of the accuracy of the similarity measurement. There are six main category patterns in this dataset set; these include normal, circular, upward trend, downward trend, upward transition, and downward transition. Each category contains 100 rows of data, with 60 data in each row. All trends were collectively referred to as “abnormal trends” with the exception of the normal trend. All the abnormal trends must be corrected, therefore, it is important to detect the abnormal trends quickly and accurately for strict control processes and good product quality. Because time series datasets have high requirements for the correctness and time complexity of the algorithms, they are a logical choice for classification experiments that analyze and study the time series classification algorithms starting from the correctness and time complexity of the results.

It was important to understand the data structure before applying the SYNDATA dataset. The synthetic control chart dataset had a labeled dataset. The given dataset *D*_*m*×*n*_ is a matrix with *m* = 600 rows and *n* = 60 columns; every 100 rows of data in 600 rows is one category with a total of six categories. In Table 3, we describe in detail the composition of the dataset. When using the SYNDATA dataset for time series classification experiments, each record is treated as a separate query sequence. Each record needs to be calculated with the remaining 599 records for cloud model similarity, then the top k largest ones are selected according to the similarity ranking, and categorized according to the group to which the k numbers belong.

**Table 3:** SYNDATA-the composition and content details of the data set.

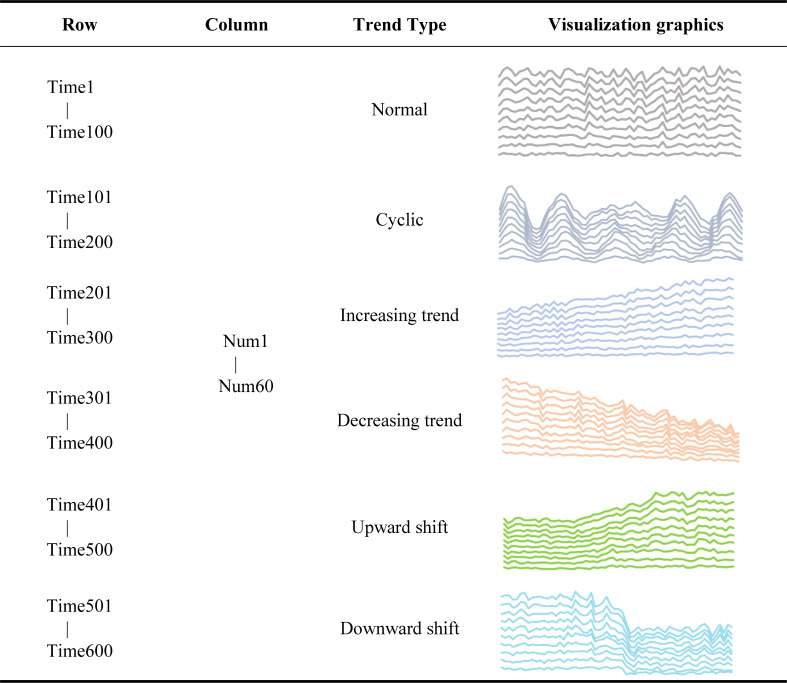

#### Detailed introduction of time series classification accuracy test

**Figure 7:**
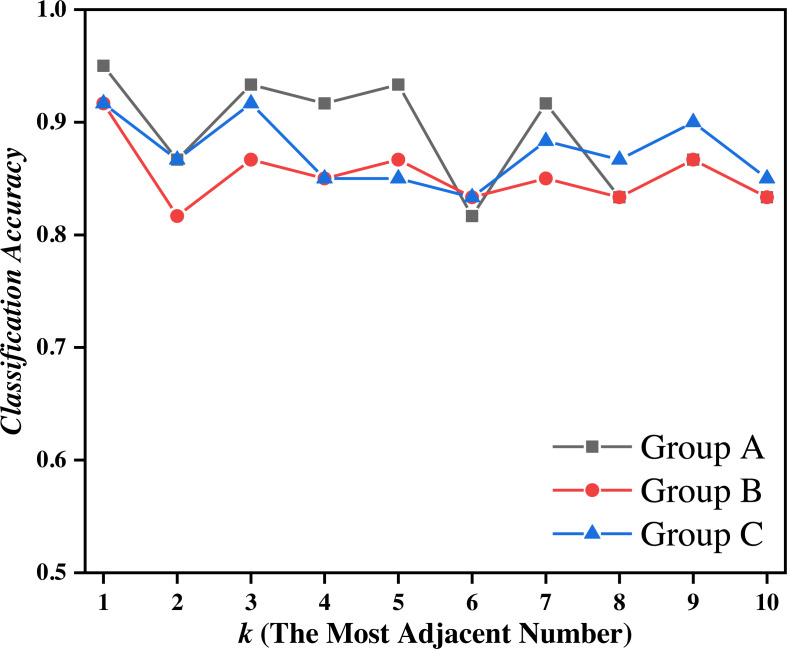
Classification accuracy of the EPTCM method under three kinds of training data with different *k* values.

**Figure 8:**
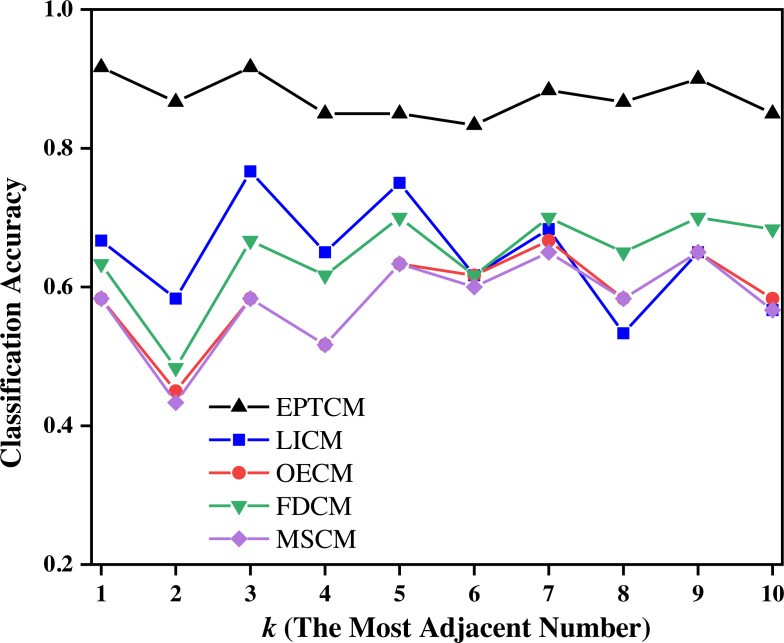
Classification accuracy of different methods on the training set of group C when *k* = 1 ∼ 10.

To verify the accuracy and rapidity of the EPTCM method in a time series classification, we used the following algorithm and the last 10 rows of data of each main category pattern were used to form the test set. Previous studies interlaced the extraction of grayscale images in order to improve the efficiency of grayscale image processing. Here, the first 90 lines of data of each main category pattern were interlaced, leaving only odd lines. After the interlaced extraction, there were only 45 lines of data of each main category in the training set. The remaining 45 lines of data in each main category were divided into three groups, which were each subdivided into three groups labeled A, B, and C. The groups of data labeled A, B, and C each contained the every “abnormal trends”. Next, the data groups, A, B, and C, were classified using the k-NN (k-nearest neighbor) (Fuchs et al., 2010; Lin et al., 2007) algorithm in the machine learning algorithm. The k-NN algorithm is easy to use and has a simple process. Every row of data in the training set and the test set can be quantitatively represented as a triangular cloud model by the reverse cloud generator, therefore, the EPTCM method can be introduced into the k-NN algorithm. The traditional k-NN algorithm determines the number of nearest neighbors of a certain eigenvalue by comparing the distances between the eigenvalues and resulting in the classification of a different eigenvalue. The EPTCM method was used to replace the relative distances between the measured eigenvalues in the traditional KNN algorithm for data classification and statistics. The structural framework of the time series classification accuracy experiment is shown in Table 4. The classification accuracy is shown in Eq. (20) and the classification results are shown in Figs. 7 and 8, and Table 5.

(20) \begin{eqnarray*}{P}_{X}= \frac{Number\,of\,correctly\,classified\,samples}{The\,total\,number\,of\,samples} \end{eqnarray*}

(21) \begin{eqnarray*}Average\,Accuracy \left( Group\,X \right) = \frac{\sum _{i=1}^{10} \left( {P}_{X} \left( k=i \right) \right) }{10} ,X=A,B,C\end{eqnarray*}

**Table 4:** Structural framework for time series classification accuracy experiments.

**Steps**	**Content**
Step 1: Calculate the feature vectors	The data in the dataset *D*_*m*×*n*_ are followed by the inverse cloud generator algorithm to obtain three numerical features for each set of data and use them as the feature vector for the *i* th record (time series):
	*C*_*i*_ = (*Ex*_*i*_, *En*_*i*_, *He*_*i*_)
	This enables the dimensionality reduction of the dataset, where 1 ≤ *i* ≤ *m*;
Step 2: Determine the training set and test set	The last 10 rows of data for each major category pattern in the *D*_*m*×*n*_ were used to form the test set. The rest of the data is used as the training set. After interlaced extraction, there are only 45 lines of data of each main category in the training set. And the remaining 45 lines of data in each main category are divided into three groups, which are grouped into three groups: A, B and C in turn. Make the three groups of data A, B and C each contain all kinds of “abnormal trends”;
Step 3: Calculate the similarity matrix	The (EPTCM, LICM, FDCM, OECM, MSCM) method is used to calculate the similarity between the feature vectors corresponding to the data in the test set and the feature vectors corresponding to the data in the training set. The resulting similarity matrix is generated:
	$Sim= \left[ \begin{array}{@{}c@{}} \displaystyle Sim \left( {C}_{Test1},{C}_{Train1} \right) \cdots Sim \left( {C}_{Test1},{C}_{Train45} \right) \\ \displaystyle \vdots \ddots \vdots \\ \displaystyle Sim \left( {C}_{Test60},{C}_{Train1} \right) \cdots Sim \left( {C}_{Test60},{C}_{Train45} \right) \end{array} \right] $
	$Sim \left( {C}_{Testi},{C}_{Trainj} \right) $ denotes the similarity between *C*_*Testi*_ and *C*_*Trainj*_ calculated using the EPTCM algorithm;
Step 4: Classification experiments are performed according to the nearest neighbor k values	First, each row of the similarity matrix $Sim \left( {C}_{Testi},{C}_{Trainj} \right) $ is sorted from largest to smallest, then the top 1 ≤ *k* ≤ 10 values of each row of the test set similarity matrix $Sim \left( {C}_{Testi},{C}_{Trainj} \right) $are selected according to the number of nearest neighbors *k*. Finally, the time series classification experiments are completed by categorizing the number of groups to which the *k* numbers belong.

**Table 5:** Average Accuracy (Eq. (21)) of different methods on various training sets when *k* = 1 ∼ 10.

***Average Accuracy***	EPTCM	LICM	OECM	FDCM	MSCM
**Group A**	0.887	0.578	0.640	0.638	0.650
**Group B**	0.853	0.607	0.533	0.602	0.532
**Group C**	0.873	0.647	0.587	0.645	0.580

## Discussion

### Simulation experimental result analysis

The similarity order of the cloud models calculated by the proposed EPTCM method is as follows: $ \left( {C}_{1},{C}_{4} \right) \gt \left( {C}_{2},{C}_{3} \right) \gt \left( {C}_{3},{C}_{4} \right) \gt \left( {C}_{1},{C}_{3} \right) \gt \left( {C}_{2},{C}_{4} \right) \gt \left( {C}_{1},{C}_{2} \right) $ (Table 2). This is consistent with the visual impression in Fig. 5. Table 2 shows that the *discrimination* of the EPTCM method for the cloud models in group A and group B was 0.11. The EPTCM method had the highest *discrimination* among the five methods, which can better identify the similarity of the cloud models and better reflect the differences between cloud models A and B. The MSCM method had a *discrimination* of 0.0932 between two groups of cloud models, which is second only to the EPTCM method. However, the measurement range of the MSCM method is very limited. Table 2 reveals that the results of the other four groups of similarity were all 0 (negative values will revert to 0), with the exception of *Sim*(*C*_1_, *C*_4_) and *Sim*(*C*_2_, *C*_3_). These results are limited and are inconsistent with human cognition. The results of the OECM method were 0.0234 for the *discrimination* between the cloud models of groups A and B. This indicates that the discriminatory ability of the OECM method is poor compared to the EPTCM method. The *discrimination* of the FDCM method was poor with results of 0.0104. It is not difficult to see from the data in Table 2 that the *Sim*(*C*_2_, *C*_4_) of the FDCM method is NAN. This is due to the limitation of its algorithm itself. The method will lose the influence of the judgment expectation on the similarity when the *En* and *He* of both clouds are equal. We can see that the cloud similarities of LICM are close to 1 and the *discrimination* is not clear. These results are obviously inconsistent with intuitive human feeling. Through the above analysis, it can be reflected that the EPTCM method proposed in this paper has certain superiority compared with the four existing methods.

### Accuracy of the experimental result analysis of time series classification

**Figure 9:**
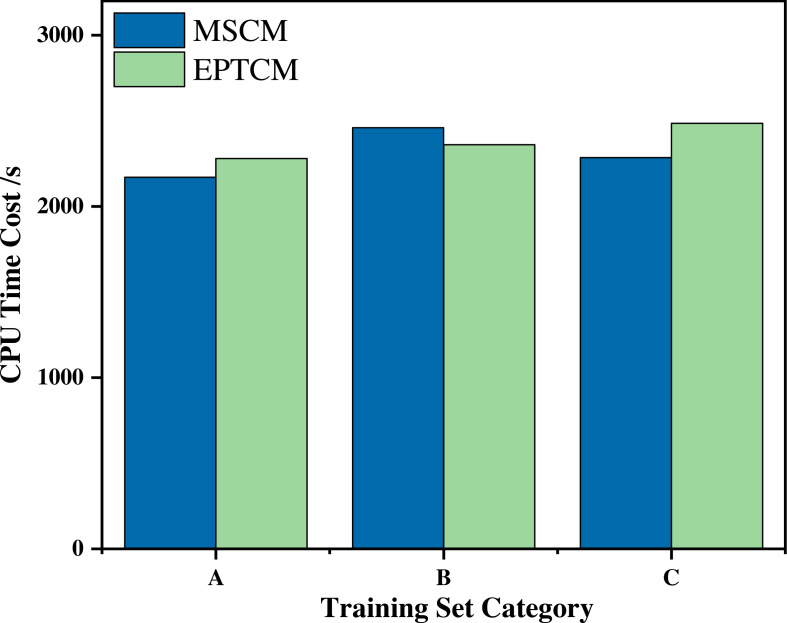
Comparison of CPU consumption time between the EPTCM method and the MSCM method at runtime.

Figure 7 shows that the proposed EPTCM method has a good classification accuracy in the time series classification experiment. When *k* was 1, 3, 4, 5, or 7, the classification accuracy of EPTCM was over 90% in the time series classification experiment with group A. According to the analysis of the data in Table 5, EPTCM achieved *Average Accuracy* of over 85% in the time series classification experiment with three training sets. Figure 8 shows the classification accuracy of each method in the range of *k* = 1 ∼ 10 when the group C training set was used in the time series classification experiments. Figure 8 clearly shows that EPTCM was the best among the five methods in terms of classification accuracy. The classification accuracy of the EPTCM method also shows good stability with the change of *k* value in the group C training set. The proposed EPTCM method has obvious advantages compared with the existing methods that exhibit low classification accuracy and poor stability. Figure 9 shows that the EPTCM method is similar to the MSCM method in CPU overhead time. However, the *Average Accuracy* of EPTCM was better than that of MSCM. Table 5 shows that the average accuracy of the EPTCM method was significantly better than that of the four existing algorithms when groups A, B, and C were used separately.

### A comparative analysis of cloud model similarity metrics

Studies by Li, Wang & Yang (2019) and Li et al. (2020) provide the evaluation metrics for cloud model similarity metrics. Table 6 shows how the EPTCM method compares with the extant methods in terms of discriminability, efficiency, stability, and interpretability. Discriminability refers to the ability of the similarity matrix to distinguish between the differences of two concepts that are not identical; efficiency refers to the time complexity of computing the similarity between two concepts; stability refers to the fact that the value of the similarity measure is constant over multiple calculations; interpretability means that the process of calculating the similarity metric is interpretable. LICM has high efficiency and stability, but it does not distinguish the differences between two concepts with the same proportion of numerical features. Additionally, considering numerical features as a vector does not reflect the relationship between the numerical features and this lacks interpretability in the calculation process. Simulation experiments also confirm its low discriminatory ability. MCM and OECM are similarity metrics based on the overlap of feature curves. They have high efficiency and stability like LICM, but they do not find the difference between two different concepts in some cases because the variation of *He* is ignored. Moreover, they are only partially interpretable due to the lack of relationship between *En* and *He*. The experimental results show that MSCM has a moderate discriminative power; it also contains an integral operation process with high time complexity and its metric results are not affected by the number of cloud drops and the number of experiments. According to the results of the simulation experiments conducted by Li et al. (2020), UDCM has good discriminative ability and theoretical interpretability. However, it retains many integral operations and is less efficient. Although the authors verify that the results are less variable compared to other algorithms, the results are not constant. The simulation experiments prove that the EPTCM method has good resolution ability and the metric results of the EPTCM method are constant and have good stability. These results are easily obtained from Algorithm 1. The EPTCM method performs a similarity metric from the shape and distance of the cloud model in both directions and has good theoretical interpretation; it also retains a small number of integral operations and has an average performance in terms of efficiency.

**Table 6:** Comparison table of cloud model similarity metrics.

	**LICM**	**FDCM**	**OECM**	**UDCM**	**MSCM**	**MCM**	**EPTCM**
**Discriminability**	Low	Low	Low	High	Medium	Low	High
**Efficiency**	High	Medium	High	Low	Medium	High	Medium
**Stability**	High	Low	High	Medium	High	High	High
**Interpretability**	Low	Medium	Medium	High	Low	Medium	High

In summary, the proposed EPTCM method has certain advantages over the existing algorithms.

## Conclusions

We proposed a comprehensive cloud model similarity measurement, which combined the PCM and the ETCM methods to consider both distance similarity and shape similarity. Then, simulation experiments and time series classification experiments were carried out using the proposed method and the existing cloud model similarity measurement methods. The simulation results show that the metric results obtained by the EPTCM method were consistent with subjective human perception, which may prove the rationality of the method. This method also has better *discrimination* than the existing classical cloud model similarity methods. The time series classification experimental results show that the proposed EPTCM method still maintains the highest classification accuracy compared with the existing classical cloud model similarity methods. The classification accuracy remained relatively stable with changing *k* values, which verifies the feasibility and effectiveness of the algorithm. In general, the EPTCM method overcomes many defects of existing methods and the experimental results confirm its feasibility and effectiveness. However, the EPTCM method still retains some complex integration operations and takes a long time to process a large amount of data. There is also no systematic way to change the values of the parameters *p* and *n* before performing the EPTCM method to obtain a better similarity measure.

Future research will address how to introduce a better fuzzy closeness method in the process of cloud model similarity calculation for the development of the triangular cloud model.

## Additional Information and Declarations

### Data Availability

The following information was supplied regarding data availability: The code is available at GitHub and Zenodo: Time-Series-Data-Processing-and-Classification-Experiment. HTTPS: https://github.com/Jianjun158/Time-Series-Data-Processing-and-Classification-Experiment.git. Jianjun158. (2023). Jianjun158/Time-Series-Data-Processing-and-Classification-Experiment: Time-Series-Data-Processing-and-Classification-Experiment (a). Zenodo. https://doi.org/10.5281/zenodo.7685091. The synthetic control chart dataset is available at: Alcock, Robert. (1999). Synthetic Control Chart Time Series. UCI Machine Learning Repository. https://doi.org/10.24432/C59G75.
